# Harnessing Nature’s Diversity: Discovering organophosphate bioscavenger characteristics among low molecular weight proteins

**DOI:** 10.1038/srep37175

**Published:** 2016-11-15

**Authors:** Reed B. Jacob, Kenan C. Michaels, Cathy J. Anderson, James M. Fay, Nikolay V. Dokholyan

**Affiliations:** 1 Department of Biochemistry and Biophysics, University of North Carolina Chapel Hill, 120 Mason Farm Rd, Campus Box 7260, 3rd Floor, Genetic Medicine Building, Chapel Hill, NC 27599, USA; 2 Department of Chemistry, University of North Carolina, 125 South Rd Kenan Rm 225, Campus Box 3290, Chapel Hill, NC 27599, USA

## Abstract

Organophosphate poisoning can occur from exposure to agricultural pesticides or chemical weapons. This exposure inhibits acetylcholinesterase resulting in increased acetylcholine levels within the synaptic cleft causing loss of muscle control, seizures, and death. Mitigating the effects of organophosphates in our bodies is critical and yet an unsolved challenge. Here, we present a computational strategy that integrates structure mining and modeling approaches, using which we identify novel candidates capable of interacting with a serine hydrolase probe (with equilibrium binding constants ranging from 4 to 120 μM). One candidate Smu. 1393c catalyzes the hydrolysis of the organophosphate omethoate (k_cat_/K_m_ of (2.0 ± 1.3) × 10^−1^ M^−1^s^−1^) and paraoxon (k_cat_/K_m_ of (4.6 ± 0.8) × 10^3^ M^−1^s^−1^), V- and G-agent analogs respectively. In addition, Smu. 1393c protects acetylcholinesterase activity from being inhibited by two organophosphate simulants. We demonstrate that the utilized approach is an efficient and highly-extendable framework for the development of prophylactic therapeutics against organophosphate poisoning and other important targets. Our findings further suggest currently unknown molecular evolutionary rules governing natural diversity of the protein universe, which make it capable of recognizing previously unseen ligands.

Effective organophosphate (OP) neutralization is a current problem facing both farmers, from agricultural poisoning, and armed forces and first responders, from chemical weapon threats^1^,^2^,^3^,^4^,^5^. Ease of dispersal, persistence of agents, and multiple intoxication paths transform agricultural OP pesticides into lethal and effective weapons^6^. Two main OP classes have been developed and weaponized, the phosphotriester G-agents, such as sarin, and the phosphonothioate ester V-agents, such as VX^6^,^7^. OPs inhibit acetylcholinesterase (AChE) by binding with high affinity to its active site. After AChE is inhibited, acetylcholine accumulates in the synaptic cleft causing bradycardia, muscle seizures, and death through asphyxiation^8^. OP AChE inhibition proceeds through OP hydrolysis, which causes the phosphorylation of the active site serine (Ser203) followed by de-alkylation at the rate of ~1.1 × 10^5^ min^−1 ^9. Due to political instabilities around the world, the chemical weapons threat has become substantial and requires the urgent development of counter-measures.

Current treatments are post-exposure and only moderately effective. One strategy of preventative measures are stoichiometric bioscavengers, proteins that compete with AChE for binding of organophosphates, thereby sequestering the organophosphates prior to AChE inhibition such as butyrylcholinesterase (BChE)^10^,^11^,^12^. However, BChE’s high production costs and large molecular weight limit the effective use of this bioscavenger^10^. A producible, low molecular weight alternative is desirable. Two possible methods in which to obtain bioscavenger candidates are, a search of known proteins looking for promiscuous activity, or using *de novo* design to engineer proteins capable of binding OPs. Recent studies by Steinkellner *et al.* have identified two proteins with previously unknown promiscuous ene-reductase activity through geometric comparison^13^. Whereas, Rajagopalan *et al.* grafts a desired amino acid structure by *de novo* design protocols to create novel serine esterases^14^.

Here, we propose to search for low molecular weight bioscavenger candidates from within the set of proteins with known 3D structures and test plausible binding of organophosphates with molecular docking (Fig. 1a). First, we apply a rigid substructure search algorithm *Erebus*^15^ to detect candidates from the protein databank mimicking the active site of AChE. Second, to predict the binding of organophosphates to the identified scaffold, we employ a molecular docking algorithm *MedusaDock*^16^ in which we account for both ligand and receptor flexibility. We choose VX as a molecular probe for the *in silico* studies due to its exceptional lethality.

Our top three results are phosphoribosyl isomerase from *Mycobacterium tuberculosis* (PDB ID 2Y85), antigen 85-A from *Mycobacterium tuberculosis* (PDB ID 1SFR), and Smu. 1393c from *Streptococcus mutans* (PDB ID 4L9A). We validate the presence of the predicted binding interactions by testing with serine hydrolase probes. The results from a fluorescence polarization study indicate that all three candidates present OP binding interactions (with equilibrium binding constants ranging from 4 to 120 μM). Of these candidates, we show that Smu. 1393c binds the probes through covalent modification of a serine mimicking AChE inhibition. We further demonstrate that Smu. 1393c can catalyze the hydrolysis of omethoate (k_cat_/K_m_ of (2.0 ± 1.3) × 10^−1^ M^−1^s^−1^) and paraoxon (k_cat_/K_m_ of (4.6 ± 0.8) × 10^3^ M^−1^s^−1^), V- and G-agent analogs respectively. Additionally, Smu. 1393c protects AChE activity when exposed to two OP simulants and represents an OP bioscavenger candidate successfully identified using our computational workflow to mine structural databases for a novel target for a specific small molecule.

## Results

### Identification of putative candidate scaffolds

**Figure 1:**
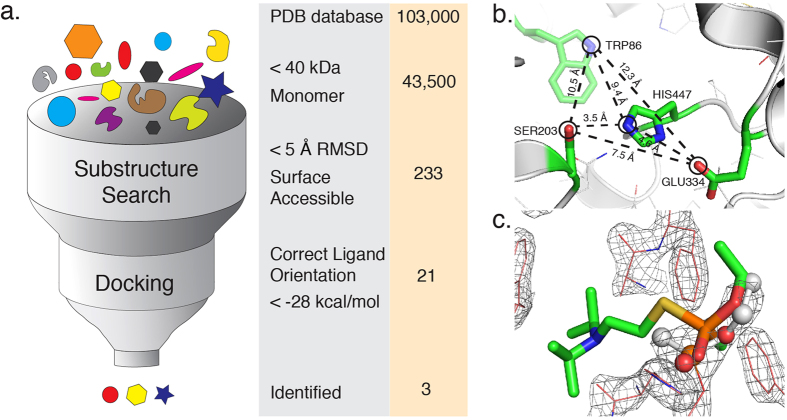
Two-stage search workflow with identified search parameters. **(a)** In this illustration we depict the computational protocol as a funnel where the various levels represent the initial population of PDB structures (top of funnel), the substructure search algorithm, *Erebus*, the docking algorithm, *MedusaDock*, and the final predicted candidates (below funnel). Alongside the illustration are two columns where we list the filter conditions at each step (first column) and the number of resulting structures for that step (second column). The search database comprises a snapshot of the PDB database from December 2014. **(b)** We show the geometric feature submitted to *Erebus,* which is comprised of four selected atoms from AChE (PDB ID 1F8U) and the connections between them to represent the geometric feature that *Erebus* searches for. **(c)** We show the validation of *MedusaDock* where the docked VX molecule lies within the electron density map of the phosphorylated serine of BChE (PDB ID 2XQF).

We first identify proteins with similar binding sites to AChE and BChE, using rigid substructure search algorithm, *Erebus*^15^ (Fig. 1a). *Erebus* algorithm rapidly extracts selected geometric features, such as inter-atomic pairwise distances, of a submitted query scaffold and then searches the PDB structures for matching features. The returned matches represent structures with a root mean square deviation (RMSD) where the query atoms and the matching result atoms fall within the desired cutoff parameter; in our case we choose a cutoff of less than five angstroms. Our query scaffold is composed of atoms selected from the active site of AChE (Fig. 1b). The number of returned results from this query is substantial (greater than 1,000) and require additional refinement, in which we apply the following three criteria: (i) molecular weight below 40 kDa to ensure a low molecular weight; (ii) monomeric form of the retrieved solutions as detailed in the PDB file; and (iii) solvent accessibility of amino acids in the retrieved hits, which is a direct assessment of whether the scaffold can be accessed by the OPs. We derive these criteria to overcome the limitations of BChE as a stoichiometric bioscavenger, namely the tetrameric structure and large molecular weight. After this refinement there are 21 protein candidates remaining (Supplementary Table 1).

### Identification of top-ranked candidates

The classic binding mode of OPs involves covalent binding of the phosphate group to an activated serine. Prior to forming covalent interactions, non-covalent interactions assist in orienting the OP for a successful serine nucleophilic attack. We screen the 21 protein candidates using a flexible docking algorithm *MedusaDock*^16^,^17^ to model non-covalent protein-ligand interactions with VX as our ligand of interest (Fig. 1a and 1c). *MedusaDock* comprises a docking step intertwined with a scoring step, where the docking step creates a ligand rotamer library and searches the best fit within the flexible side chain rotamer library, using the *MedusaScore* force field^16^,^17^. We select the final candidates through ranking the 21 candidates by best-predicted binding energy (Supplementary Table 1). We find this method of ranking reveals a natural division between the candidates specifically: (i) an *Erebus* RMSD less than 4 Å; (ii) an average ligand RMSD less than 8 Å; and (iii) a *MedusaDock* predicted binding energy less than −28 kcal/mol. The top resulting candidates are antigen 85-A (PDB ID 1SFR) and phosphoribosyl isomerase (PDB ID 2Y85) from *Myobacterium tuberculosis* and Smu. 1393c from *Streptococcus mutans* (PDB ID 4L9A).

**Figure 2:**
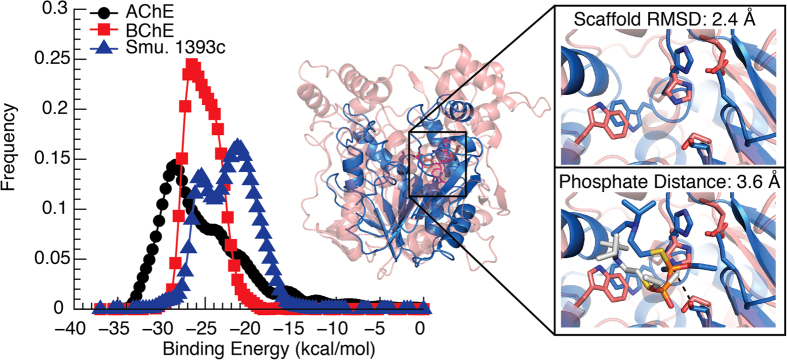
Predicted Binding Energy distributions, structural overlays, and active site comparisons of candidate Smu. 1393c. (left) We show that Smu. 1393c (PDB ID 4L9A) (blue curve) is predicted to have binding interactions similar to control AChE (PDB ID 1F8U) and BChE (PDB ID 2XQF) (black and red curves respectively), through the overlap of predicted binding energy distributions of the organophosphate VX. (middle) We show Smu. 1393c’s lower molecular weight by structurally aligning Smu. 1393c (blue) with a molecular weight of ~33 kDa to a monomer of BChE (red) with a molecular weight of ~84 kDa. (right-top) We show that the predicted residues of Smu. 1393c (blue) align with the active site of BChE (red) in the absence of VX. (right-bottom) We show that the lowest energy pose of VX docked with Smu. 1393c (blue) and BChE (gray) place the phosphate in the correct orientation to irreversibly bind the active serine.

To evaluate predicted binding modes of identified candidates, we compare predicted binding energy distributions of organophosphate complexes with identified candidates to that of AChE and BChE. The greater the overlap between these distributions means the greater the probability of mimicking AChE OP interactions (Fig. 2 and Supplementary Fig. 1). From docking simulations of all three candidates we find that the distance between the VX phosphate and predicted serine are less than 4 Å. This distance places the VX phosphate in a position for a serine nucleophilic attack. Additionally, the lowest predicted binding energy for each of our candidates’ is within a 6 kcal/mol difference to the value of −35.3 kcal/mol calculated for AChE (Supplementary Table 1). This difference indicates that our three candidates show predicted characteristics similar to AChE.

### OP binding to top candidates

**Figure 3:**
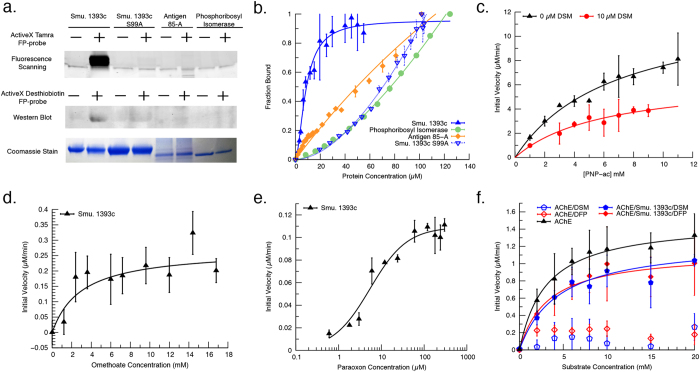
Experimental results of candidate OP binding using serine hydrolase probes, AChE protection assays, and catalytic activity. **(a)** We show that candidate Smu. 1393c (PDB ID 4L9A) is covalently labeled by two serine hydrolase probes, whereas Smu. 1393c S99A, antigen 85-A (PDB ID 1SFR), and phosphoribosyl isomerase (PDB ID 2Y85) are not covalently labeled. For each serine hydrolase probe we include a parallel coomassie stained SDS-PAGE gel showing the presence of protein candidates. **(b)** We show that candidates Smu. 1393c (blue triangle), antigen 85-A (orange), and phosphoribosyl isomerase (green) interact with the serine hydrolase probe as predicted and confirm the diminished binding of Smu. 1393c S99A (inverted blue triangle). **(c)** We show the activity of Smu. 1393c against *p-*nitrophenyl acetate uninhibited (black triangles) and inhibited with demeton-S-methyl (DSM) (red circles). **(d,e)** We show that Smu. 1393c catalyzes the hydrolysis of organophosphates omethoate (d) and paraoxon (e) with the initial velocity plot fitted to Michaelis-Menten equation. **(f)** We show that Smu. 1393c protects AChE activity with (i) AChE uninhibited positive control (black), (ii) protected AChE activity from demeton-S-methyl (blue filled) and diisopropylfluorophospate (red filled), and (iii) AChE inhibited negative controls, demeton-S-methyl (blue outline) and diisopropylfluorophospate (red outline). All experiments were run in triplicate.

We validate predicted candidate similarity to AChE by using serine hydrolase probes, specifically ActiveX Tamra fluorophosphonate serine hydrolase probe (TFP-probe) and ActiveX Desthiobiotin fluorophosphonate serine hydrolase probe (DFP-probe) (Supplementary Fig. 2). These probes covalently phosphorylate, thereby labeling an active serine through a similar mechanism as organophosphates (Supplementary Fig. 3). The labeled serine is detectable via fluorescent gel (TFP-probe) or western blot (DFP-probe). A clearly defined band was present for Smu. 1393c, indicating positive phosphorylation (Fig. 3a and Supplementary Fig. 4). We did not detect a band for phosphoribosyl isomerase or antigen 85-A, suggesting no covalent binding though non-covalent interactions may still be present.

To quantify OP binding and check for non-covalent interactions, we use fluorescent polarization experiments utilizing the TFP-probe as substrate (Fig. 3b and Supplementarty Fig. 5). We find that Smu. 1393c has low micromolar binding (K_d_ = 4.17 ± 0.08 μM). Though candidates’ antigen 85-A and phosphoribosyl isomerase show no covalent binding, there are non-covalent binding interactions with K_d_’s of 20.9 ± 1.6 μM and 120 ± 10 μM, respectively. All three candidates display binding interactions that validate the predictions from our computational workflow.

### OP covalent binding to Smu. 1393c active site

To confirm that the location of the phosphorylation is as predicted from our computational workflow, we substitute serine (Ser99) to alanine in the Smu. 1393c’s predicted active site and perform labeling with both TFP-probe and DFP-probe and fluorescent polarization experiments with TFP-probe. A visible band indicating phosphorylation was not present in the Ser99 mutation, thereby covalent binding was indeed eliminated from both the fluorescent gel and the western blot analysis (Fig. 3a). In addition, the removal of Ser99 drastically increases the K_d_ to greater than 300 μM (Fig. 3b), suggesting that Ser99 plays an essential role in OP binding.

### Smu. 1393c catalytic activity

The literature indicates that Smu. 1393c has catalytic activity against *p*-nitrophenyl acetate (PNP-ac)^18^. We determine the activity rate of Smu. 1393c aided hydrolysis of PNP-ac to be k_cat_/K_m_ of 6.3 ± 1.3 M^−1^s^−1^ (Fig. 3c, Table 1). Exposure to V-agent analog demeton-S-methyl (DSM) inhibits the PNP-ac hydrolysis activity with a K_i_ of 10.64 ± 1.9 μM. Using a double reciprocal plot we find that DSM inhibits Smu. 1393c in a non-competitive manner (Supplementary Fig. 6), suggesting a potential second site that when bound has direct ramifications on activity.

### Smu. 1393c protection of AChE

The inhibition of Smu. 1393c by organophosphate DSM indicates a protein/ligand interaction. We explore the ability of Smu. 1393c to protect AChE from inhibition through a quantitative set of activity assays, modified from Cherny *et al.*, designed to gauge the amount of AChE activity maintained after exposure to two OP simulates^19^. Specifically, we use demeton-S-methyl (DSM) as a V-agent analog and diisopropylfluorophosphate (DFP) as a G-agent analog (Fig. 3f, Supplementary Fig. 2, and Supplementary Table 2). All measurements are expressed as relative activity to uninhibited AChE. We observe that Smu. 1393c protects AChE from full inhibition, preserving 84.4 ± 4.1% of activity for DSM and 76 ± 10% for DFP. Additionally, we find no significant change between the K_m_ of active AChE (3.05 ± 0.79 mM) and inhibited AChE (4.43 ± 0.63 mM for DSM and 3.2 ± 1.3 mM for DFP). These results indicate that Smu. 1393c may act as an OP bioscavenger to protect AChE from inhibition.

### Smu. 1393c activity against OPs

To investigate whether Smu. 1393c functions catalytically against OPs, we first use DSM as a V-agent analog. We find that Smu. 1393c shows unanticipated catalytic activity against DSM but are unable to complete the kinetic assays as the concentrations required to reach kinetic saturation exceed our supply. Additionally, to fully test the hydrolysis of the G-agent analog DFP requires an experimental setup unsupported in our laboratory. These reasons necessitate our change from DFP and DSM to paraoxon and omethoate as G- and V-agent analogs respectively (Supplementary Fig. 2). After a series of kinetic assays we find that Smu. 1393c catalyzes the hydrolysis of omethoate with a k_cat_/K_m_ of (2.0 ± 1.3) × 10^−1^ M^−1^s^−1^ (Fig. 3d, Table 1). When we test paraoxon hydrolysis the activity level is below the sensitivity of our instruments. Intriguingly, when we express Smu. 1393c with zinc and then test activity we see hydrolysis of paraoxon with a k_cat_/K_m_ of (4.6 ± 0.8) × 10^3^ M^−1^s^−1^ (Fig. 3e, Table 1) and a loss of detectable omethoate activity. These findings suggest two different catalytic mechanisms. Interestingly, when we perform the same tests with Smu. 1393c S99A mutant we find no activity for either omethoate or paraoxon, suggesting that Ser99 is crucial in both mechanisms.

## Discussion

OP poisoning is a worldwide problem with an estimated 3,000,000 people exposed yearly with up to 200,000 fatalities^20^,^21^. The availability of cheap and effective therapeutics becomes a necessity, as developing countries use OP pesticides in agriculture^22^. Additional concern lies in exposure to the more toxic variety of OPs, namely OP chemical nerve agents. These chemical weapons are cheap, deadly and present a viable threat in warfare or terrorist attack scenarios. The current preventative measures employed by military personnel include protective equipment and rigorous decontamination procedures^23^. These measures are only effective if *a priori* intelligence of an exposure situation allows proper implementation. What is needed is a molecular therapeutic designed to prevent AChE inhibition such as a stoichiometric bioscavenger, to competitively bind OPs, or a catalytic bioscavenger, to degrade OP chemicals. BChE, the current stoichiometric bioscavenger candidate, has a high molecular weight and high cost of production^10^, both of which limit its effectiveness.

Here we present three low molecular weight, producible bioscavenger candidates, antigen 85-A and phosphoribosyl isomerase from *Mycobacterium tuberculosis* and Smu. 1393c from *Streptococcus mutans.* Analysis of the structures reveals both antigen 85-A and Smu. 1393c have a core structure of an α/β hydrolase, and fall within the same fold class as AChE and BChE, but have less than five percent identity with either AChE or BChE^18^. Phosphoribosyl isomerase is unique among the three candidates with a core structure of a (β/α)_8_-barrel scaffold^24^. These candidates are predicted to form non-covalent interactions with OPs and are experimentally verified using serine hydrolase probes with equilibrium binding constants between 4 and 120 μM.

We establish that Smu. 1393c has the potential to protect AChE from inhibition. To be considered an OP bioscavenger there remains further investigation to determine the immunological effects from introducing such a foreign protein into the immune system. Additionally we show that Smu. 1393c has activity against OP omethoate (k_cat_/K_m_ of (2.0 ± 1.3) × 10^−1^ M^−1^s^−1^) in the absence of zinc but is active against paraoxon (k_cat_/K_m_ of (4.6 ± 0.8) × 10^3^ M^−1^s^−1^) when zinc is present. Examining the crystal structure of Smu. 1393c, we find a preponderance of histidine residues in the same cleft where the active serine (Ser99) is located that we suspect may aid in metal coordination (Supplementary Fig. 7). This hypothesis will be the subject of further investigations to understand the exact mechanism behind Smu. 1393c’s hydrolysis activity. The level of activity that Smu. 1393c possesses falls far below that of metal-binding proteins, such as human paraoxonase-1 or organophosphate hydrolase. For Smu. 1393c to be a competitive organophosphate will require redesign of the hydrolysis mechanism.

By identifying these bioscavenger candidates we demonstrate our computational workflow (Supplementary Fig. 8) which combines two successful algorithms; (i) *Erebus*^15^, a structural database search algorithm to identify molecules with substructures matching the query template; and (ii) *MedusaDock*^16^, a small molecule docking algorithm to predict the probability of interaction (see Supplementary text). We believe that with further development and automation, this workflow can be extended into applications benefitting both the pharmaceutical industry and the biotech community. Specifically, by aiding in drug adverse side-effect studies, molecular therapeutics, epitope vaccine design, drug repurposing, and protein sensor design.

## Materials and Methods

### Search for Novel Scaffolds

To effectively search the PDB database (www.rcsb.com), currently containing over 100,000 structures (December 2014), we select all proteins with less than 350 residues and less than four subunits. We search this subset using the rigid substructure search algorithm *Erebus*^15^ by submitting a target scaffold defined by a group of atoms with known coordinates. The active site target scaffold we use is constructed using the hydroxyl oxygen from Ser203, the carboxylic oxygen from Glu334, the *tele* nitrogen from His447, all from the catalytic triad as oriented in the crystal structure of AChE (PDB ID 1F8U). We also include the indolic nitrogen from Trp86 to describe the binding pocket diameter. We use the default settings and set the RMSD cutoff at five angstroms. The final input scaffold consists of atoms with associated Cartesian coordinates formatted in the PDB style. Our starting query is below:

ATOM 1543 OG SER A 203 116.605 106.967-138.509 1.00 53.76 O

ATOM 3355 ND1 HIS A 447 116.432 102.538-135.187 1.00 56.88 N

ATOM 2485 OE1 GLU A 334 114.554 100.680-134.867 1.00 62.46 O

ATOM 634 NE1 TRP A 86 125.948 105.322-133.913 1.00 37.58 N

Additionally, the computational cost of *Erebus* is minimal, taking a few minutes to hours, depending on the number of atoms in the search query, to search the entire structural database. For our query, the structural database was searched in ~1.5 hours.

### Docking Simulations

We perform redocking of VX with BChE (PDB ID 2XQF) (Fig. 1c) as a control to benchmark the docking method and to obtain the reference distribution of docking scores for the native complex as well as with AChE (PDB ID 1F8U). The input files for *MedusaDock*^25^ consist of (1) the target protein in PDB format, (2) the ligand of interest in a Mol2 format, and (3) the center for the search sphere marked by a small molecule or atom in the Mol2 format. To screen the 21 protein candidates we dock each candidate 100 times, each docking attempt is independent and includes ligand conformational sampling and simulated annealing. We select the top three candidates and dock them 1,000 times to extract a predicted binding energy distribution to compare to the reference distributions, where the frequency histogram plotted is the number of docked poses that fall within that bin. The computational cost of one *MedusaDock* calculation is on average between 4–6 minutes. To achieve results in a timely manner we distributed the 1,000 docking experiments over the killdevil computer cluster at the University of North Carolina Chapel Hill.

### Protein Expression and Purification

We optimize the candidate protein gene sequences for expression in *E. coli* with an added poly-his tag and TEV protease site at the N-termini and clone the genes into the pET14B vector for expression in the *E. coli* strain BL21 (DE3) pLysS. We grow the cells in both standard LB (Smu. 1393c and phosphoribosyl isomerase) and super broth (antigen 85-A). For the case of the catalytically active Smu. 1393c, we grow the cells in standard LB with 0.5 mM ZnCl_2_. In all cases, we induce expression with 1 mM Isopropyl β-D-1-thiogalactopyranoside (IPTG) at 25 °C overnight for phosphoribosyl isomerase and Smu. 1393c, and 72 hours at 18 °C for antigen 85-A. The collected cells are stored at −80 °C until use.

To purify we defrost the cell pellets on ice and resuspend in buffer A (50 mM phosphate pH 7.4, 500 mM NaCl, 40 mM imidazole, 5 mM βME, and 0.1 μM Pepstatin A). We use sonication to lyse the cells and centrifuge them in a Beckman J-20 rotor at 15,000 g for 2 hours. We collect and filter the supernatant through a 0.22 μm filter before loading on a 5 mL HisTrap FF column from GE. We elute the protein with a gradient to buffer B (50 mM phosphate pH 7.4, 150 mM NaCl, 1 M imidazole). We then dialyze the proteins against 20 mM Tris pH 7.4 and elute from a HiTrap Q FF column from GE. We perform all column runs using a BioRad BioLogic low-pressure chromatography machine.

### Fluorophosphonate labeling and fluorescence polarization

We label each protein with both ActiveX Tamra fluorophosphonate serine hydrolase probe (TFP-probe) for fluorescent gel scanning, and ActiveX Desthiobiotin fluorophosphonate serine hydrolase probe (DFP-probe) to show binding via western blot. Both probes are purchased from Thermo Scientific. We follow the provided protocol with minor modifications. We dilute the probe into a 2 μM stock solution and calculate the necessary ratio of 1:30 to be 167 nM of probe to 5 μM protein in a 20 μL reaction. We incubate each protein sample with the probes for 15–30 minutes at room temperature. The reaction is quenched by adding 5 μL of SDS-PAGE loading dye (200 mM Tris pH 6.8, 10 mM βME 10% SNS, 30% glycerol and bromophenol blue) and boiled for 5 minutes. We analyze each sample by fluorescent gel scanning or western blot. As a negative control we include for each protein a sample with no probe.

We perform fluorescence polarization using the TFP-probe as substrate. We dilute the TFP-probe to a 200 nM concentration and titrate the given protein measuring the change in polarization until saturation. We fit the obtained data first to a one site-specific binding model, 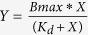, and then to a one site-specific binding model with Hill slope, 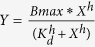. There is no substantial difference between the fits for phosphoribosyl isomerase, antigen 85-A, and Smu. 1393c S99A. We find that the one site-specific binding model with Hill coefficient produces a better fit for Smu. 1393c (Hill coefficient = 2.0 ± 0.06).

### AChE protection assays

We purchase lyophilized *Electrophorus electricus* AChE, demeton-S-methyl, and diisopropylfluorophosphate from Sigma Aldrich. We run a modified protocol from Cherny *et al.*^19^, where standard Ellman assays recover kinetic parameters^26^ and determine the ability of Smu. 1393c to protect AChE from the effects of OP simulants. We incubate equal amounts of Smu. 1393c and OP simulant (final concentration 500 nM) at 20 °C for 30 minutes. We add AChE to each sample and incubate at 20 °C for 60 minutes. As a detectable marker we add 500 μM of the Ellman reagent 5,5-dithio-bis-(2-nitrobenzoic acid) (DTNB) to the samples. We vary the amount of acetylthiolcholine (0–20 mM) and bring each sample to a volume of 50 μL using buffer (200 mM NaCl, 20 mM TRIS at pH 7.4). Each assay is performed in triplicate. We plot the kinetic parameters, obtained using SpectraMax M3 plate reader, and fit them to the Michaelis-Menton kinetic equation.

### Smu. 1393c catalytic assays

We verify the catalytic activity of Smu. 1393c by observing the absorbance of the *p*-nitrophenyl group detectable at 405 nm marking the hydrolysis of *p-*nitrophenyl acetate (PNP-ac). We also assay the inhibition potential of demeton-S-methyl. We plot and fit the kinetic parameters to the Michaelis-Menton kinetic equation and calculate the inhibition constant by 
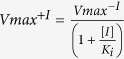
. We determine the activity of Smu. 1393c against both paraoxon and omethoate using absorbance. For paraoxon we detect the *p*-nitrophenyl group at 405 nm. To monitor the omethoate hydrolysis we use an Ellman assay^26^. We perform each assay in triplicate investigating paraoxon concentrations between 0–300 μM and omethoate concentrations between 0–18 mM. Each assay is brought to 1000 μL with 50 mM bis-tris-propane pH 7.4. We initiate the catalytic reaction by adding Smu. 1393c for a final concentration of 72 nM for paraoxon, and 5 μM for omethoate. Additionally, we perform negative controls without Smu. 1393c. We analyze the data by subtracting the negative controls from the calculated initial velocities. We plot and fit the kinetic parameters to the Michaelis-Menton kinetic equation.

## Additional Information

**How to cite this article**: Jacob, R. B. *et al.* Harnessing Nature’s Diversity: Discovering organophosphate bioscavenger characteristics among low molecular weight proteins. *Sci. Rep.* **6**, 37175; doi: 10.1038/srep37175 (2016).

**Publisher’s note:** Springer Nature remains neutral with regard to jurisdictional claims in published maps and institutional affiliations.

## Supplementary Material

Supplementary Information

## Figures and Tables

**Table 1:** Kinetic parameters for Smu. 1393c with substrate PNP-ac, PNP-ac and inhibitor DSM, paraoxon and omethoate.

	k_cat_ (s^−1^) (10^−3^)	K_m_ (mM)	k_cat_/K_m_ (M^−1^s^−1^)
PNP-ac	42 ± 4	6.7 ± 1.3	6.3 ± 1.3
PNP-ac + DSM	22 ± 3	6.0 ± 1.9	3.6 ± 1.3
Paraoxon*	25 ± 0.9	(5.5 ± 1) × 10^−3^	(4.6 ± 0.8) × 10^3^
Omethoate	0.52 ± 0.09	2.6 ± 1.6	0.2 ± 0.13

^*^Smu. 1393c expressed with zinc.
