# Corrigendum: Harnessing Nature’s Diversity: Discovering organophosphate bioscavenger characteristics among low molecular weight proteins

**DOI:** 10.1038/srep42832

**Published:** 2017-02-17

**Authors:** Reed B. Jacob, Kenan C. Michaels, Cathy J. Anderson, James M. Fay, Nikolay V. Dokholyan

Scientific Reports 6: Article number: 3717510.1038/srep37175; published online: 11 15 2016; updated: 02 17 2017

The Acknowledgements section in this Article is incomplete.

“The authors acknowledge David Mowrey, Marino Convertino, and Onur Dagliyan for their assistance in logic discussions. This project is funded by DTRA grant HDTRA-1-12-C-0074. The plasmid for phosphoribosyl isomerase was a gift from the lab of Dr. Matthias Wilmanns”.

Should read:

“The authors acknowledge David Mowrey, Marino Convertino, and Onur Dagliyan for their assistance in logic discussions. This project is funded by DTRA grant HDTRA-1-12-C-0074. The plasmid for phosphoribosyl isomerase was a gift from the lab of Dr. Matthias Wilmanns. We also acknowledge Dr. Yazhong Tao for his assistance with cloning and the western blot shown in Figure 3a and Supplementary Figure 4”.

