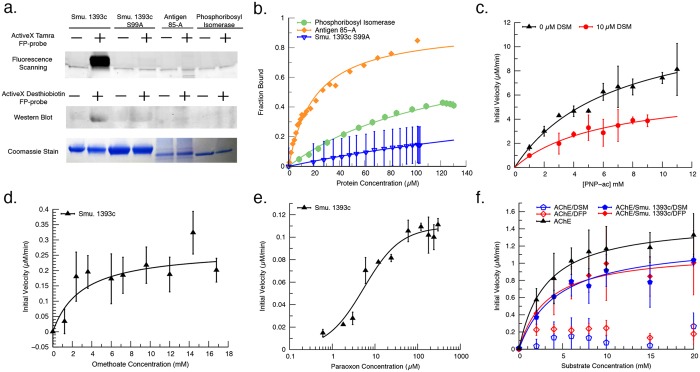# Corrigendum: Harnessing Nature’s Diversity: Discovering organophosphate bioscavenger characteristics among low molecular weight proteins

**DOI:** 10.1038/srep46727

**Published:** 2017-04-28

**Authors:** Reed B. Jacob, Kenan C. Michaels, Cathy J. Anderson, James M. Fay, Nikolay V. Dokholyan

Scientific Reports 6: Article number: 3717510.1038/srep37175; published online: 11 15 2016; updated: 04 28 2017

The original version of this Article contained errors caused by a mistake in the analysis of the fluorescent polarization experimental data. This mistake did not affect the identification of bioscavengers of organophosphates.

As a result, in the Abstract,

“Here, we present a computational strategy that integrates structure mining and modeling approaches, using which we identify novel candidates capable of interacting with a serine hydrolase probe (with equilibrium binding constants ranging from 4 to 120 μM)”.

now reads:

“Here, we present a computational strategy that integrates structure mining and modeling approaches, using which we identify novel candidates capable of interacting with a serine hydrolase probe (with equilibrium binding constants ranging from 20 to 120 μM)”.

In the Introduction section,

“The results from a fluorescence polarization study indicate that all three candidates present OP binding interactions (with equilibrium binding constants ranging from 4 to 120 μM)”.

now reads:

“The results from a fluorescence polarization study indicate that all three candidates present OP binding interactions (with equilibrium binding constants ranging from 20 to 120 μM)”.

In the Results section under subheading ‘OP binding to top candidates’,

“To quantify OP binding and check for non-covalent interactions, we use fluorescent polarization experiments utilizing the TFP-probe as substrate (Fig. 3b and Supplementarty Fig. 5). We find that Smu. 1393c has low micromolar binding (K_d_ = 4.17 ± 0.08 μM). Though candidates’ antigen 85-A and phosphoribosyl isomerase show no covalent binding, there are non-covalent binding interactions with K_d_’s of 20.9 ± 1.6 μM and 120 ± 10 μM, respectively. All three candidates display binding interactions that validate the predictions from our computational workflow”.

now reads:

“To quantify OP binding and check for non-covalent interactions, we use fluorescent polarization experiments utilizing the TFP-probe as substrate (Fig. 3b and Supplementary Fig. 5). Though candidates’ antigen 85-A and phosphoribosyl isomerase show no covalent binding, there are non-covalent binding interactions with K_d_’s of 20.9 ± 1.6 μM and 120 ± 10 μM, respectively. All three candidates display binding interactions that validate the predictions from our computational workflow”.

In the Discussion section,

“These candidates are predicted to form non-covalent interactions with OPs and are experimentally verified using serine hydrolase probes with equilibrium binding constants between 4 and 120 μM”.

now reads:

“These candidates are predicted to form non-covalent interactions with OPs and are experimentally verified using serine hydrolase probes with covalent medication or equilibrium binding constants between 20 and 120 μM”.

In the Materials and Methods section under subheading ‘Fluorophosphonate labeling and fluorescence polarization’,

“There is no substantial difference between the fits for phosphoribosyl isomerase, antigen 85-A, and Smu. 1393c S99A. We find that the one site-specific binding model with Hill coefficient produces a better fit for Smu. 1393c (Hill coefficient = 2.0 ± 0.06)”.

now reads:

“There is no substantial difference between the fits for phosphoribosyl isomerase, antigen 85-A, and Smu. 1393c S99A”.

In Figure 3b, data normalized to the highest point obtained was used rather than the calculated saturation point for a true fraction bound. In addition, the Smu. 1393c wild type data has now been removed as the binding reaction does not reach equilibrium conditions due to the covalent modifications. The correct Figure 3 appears above as Figure 1.

The legend of Figure 3b,

“We show that candidates Smu. 1393c (blue triangle), antigen 85-A (orange), and phosphoribosyl isomerase (green) interact with the serine hydrolase probe as predicted and confirm the diminished binding of Smu. 1393c S99A (inverted blue triangle)”.

now reads:

“We show that candidates antigen 85-A (orange), and phosphoribosyl isomerase (green) interact with the serine hydrolase probe as predicted and confirm the minimal binding interaction of Smu. 1393c S99A (inverted blue triangle).”

In Figure S5, the Smu. 1393c wild type data has now been removed as the binding reaction does not reach equilibrium conditions due to the covalent modifications.

The legend of Figure S5,

“**Fluorescence polarization experiment**. In this figure we show that candidates Smu. 1393c (blue triangle), antigen 85-A (orange), and phosphoribosyl isomerase (green) interact with the serine hydrolase probe as predicted and confirm the diminished binding of Smu. 1393c S99A (inverted blue triangle)”.

now reads:

“**Fluorescence polarization experiment**. In this figure we show that candidates antigen 85-A (orange), and phosphoribosyl isomerase (green) interact with the serine hydrolase probe as predicted and confirm the minimal binding interactions of Smu. 1393c S99A (inverted blue triangle)”.

These errors have been corrected in the PDF and HTML versions of the Article, as well as the Supplementary Information file that now accompanies the Article.

## Figures and Tables

**Figure 1:**