# Supplementary material for: Harnessing Nature’s Diversity: Discovering organophosphate bioscavenger characteristics among low molecular weight proteins
Source: Sci Rep. 2016 Nov 15;6:37175. doi: 10.1038/srep37175 (PMC5109037; doi:10.1038/srep37175)
Supplement: Supplementary Information [file srep37175-s1.pdf]

Harnessing Nature's Diversity: Discovering organophosphate bioscavenger characteristics among low molecular weight proteins

Reed B. Jacob[a], Kenan Michaels[b], Cathy Anderson[b], James Fay[a], Nikolay V. Dokholyan[a,c]

[a] Department of Biochemistry and Biophysics, University of North Carolina Chapel Hill, 120 Mason Farm Rd, Campus Box 7260, 3rd Floor, Genetic Medicine Building, Chapel Hill, NC 27599
[b] Department of Chemistry, University of North Carolina Chapel Hill, Campus Box 3290 Chapel Hill, NC 27599
[c.] Nikolay V. Dokholyan, 120 Mason Farm Rd, Campus Box 7260, 3rd Floor, Genetic Medicine Building, Chapel Hill, NC 27599; (919) 843-2513; dokh@med.unc.edu

Keywords: organophosphate bioscavenger, chemical weapons, structure mining.

Supplementary Material

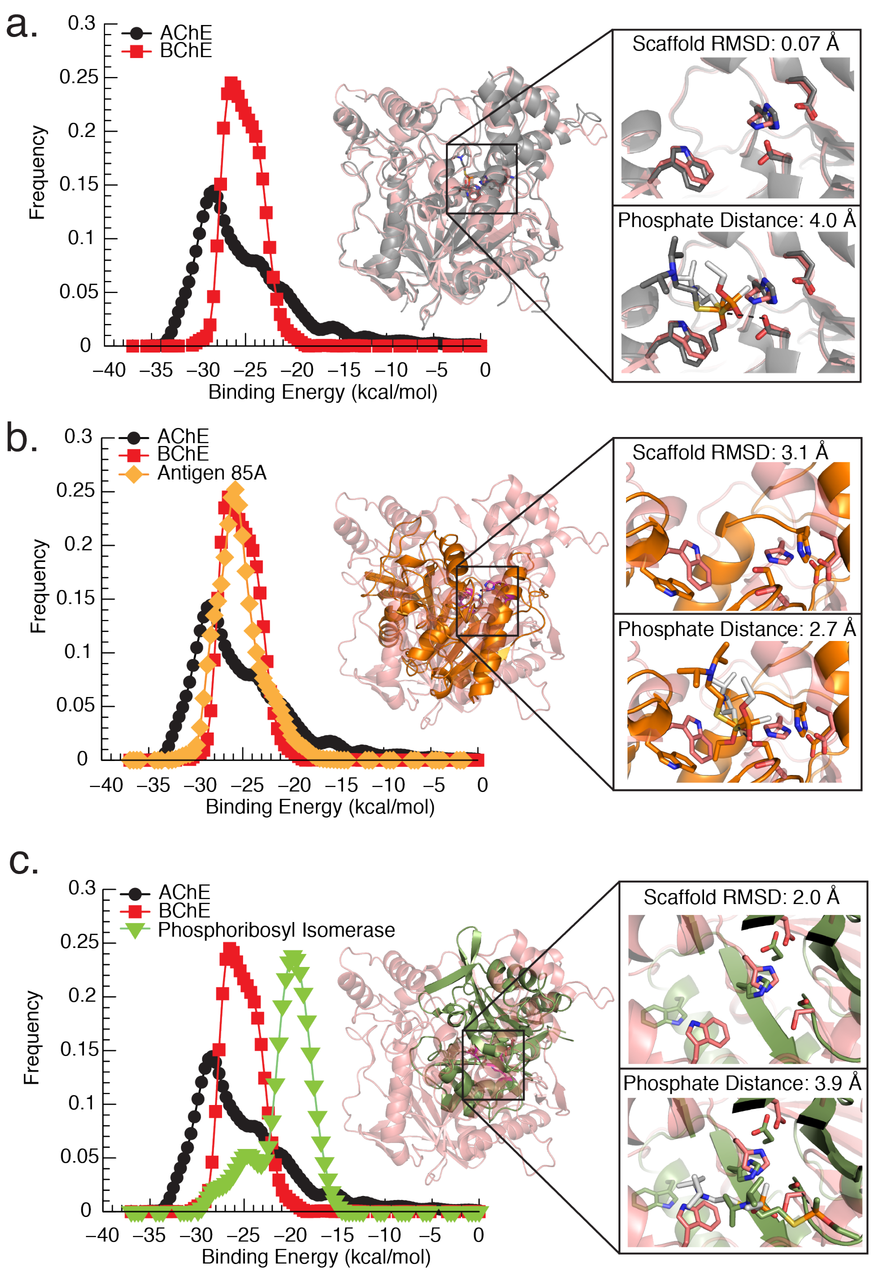

Figure S1. Predicted Binding Energy distributions, structural overlays, and active site comparisons of candidate proteins. (a) (left) We show the predicted binding energy distributions of VX agent docked in the binding site of positive controls BChE (PDB ID 2XQF) and AChE (PDB ID 1F8U) (red and black curves respectively). **(middle)** We show that the control proteins show a similar size (~84 kDa) by the structural overlay of AChE (gray) and BChE (red). **(right-top)** We show the similarity of both AChE (gray) and BChE (red) active sites in the absence of VX. **(right-bottom)** We show that the lowest energy pose of VX docked with AChE (black) and BChE (gray) place the phosphate in the correct orientation to irreversibly bind the active serine. **(b)**

(left) We show that antigen 85-A (PDB ID 1SFR) (orange curve) is predicted to have binding interactions similar to control AChE and BChE (black and red curves respectively), through the overlap of predicted binding energy distributions of the organophosphate VX. **(middle)** We show antigen 85-A's lower molecular weight by structurally aligning antigen 85-A (orange) with a molecular weight of ~35 kDa to BChE (red) with a molecular weight of ~84 kDa. **(right-top)** We show that the predicted residues of antigen 85-A (orange) align with the active site of BChE (red) in the absence of VX. **(right-bottom)** We show that the lowest energy pose of VX docked with antigen 85-A (orange) and BChE (gray) place the phosphate in the correct orientation to irreversibly bind the active serine**. (c) (left)** We show that phosphoribosyl isomerase (PDB ID 2Y85) (green curve) is predicted to have binding interactions similar to control AChE and BChE (black and red curves respectively), through the overlap of predicted binding energy distributions of the organophosphate VX. **(middle)** We show phosphoribosyl isomerase's lower molecular weight by structurally aligning phosphoribosyl isomerase (green) with a molecular weight of ~25 kDa to BChE (red) with a molecular weight of ~84 kDa. **(right-top)** We show that the predicted residues of phosphoribosyl isomerase (green) align with the active site of BChE (red) in the absence of VX. **(right-bottom)** We show that the lowest energy pose of VX docked with phosphoribosyl isomerase (green) and BChE (gray) place the phosphate in the correct orientation to irreversibly bind the active serine.

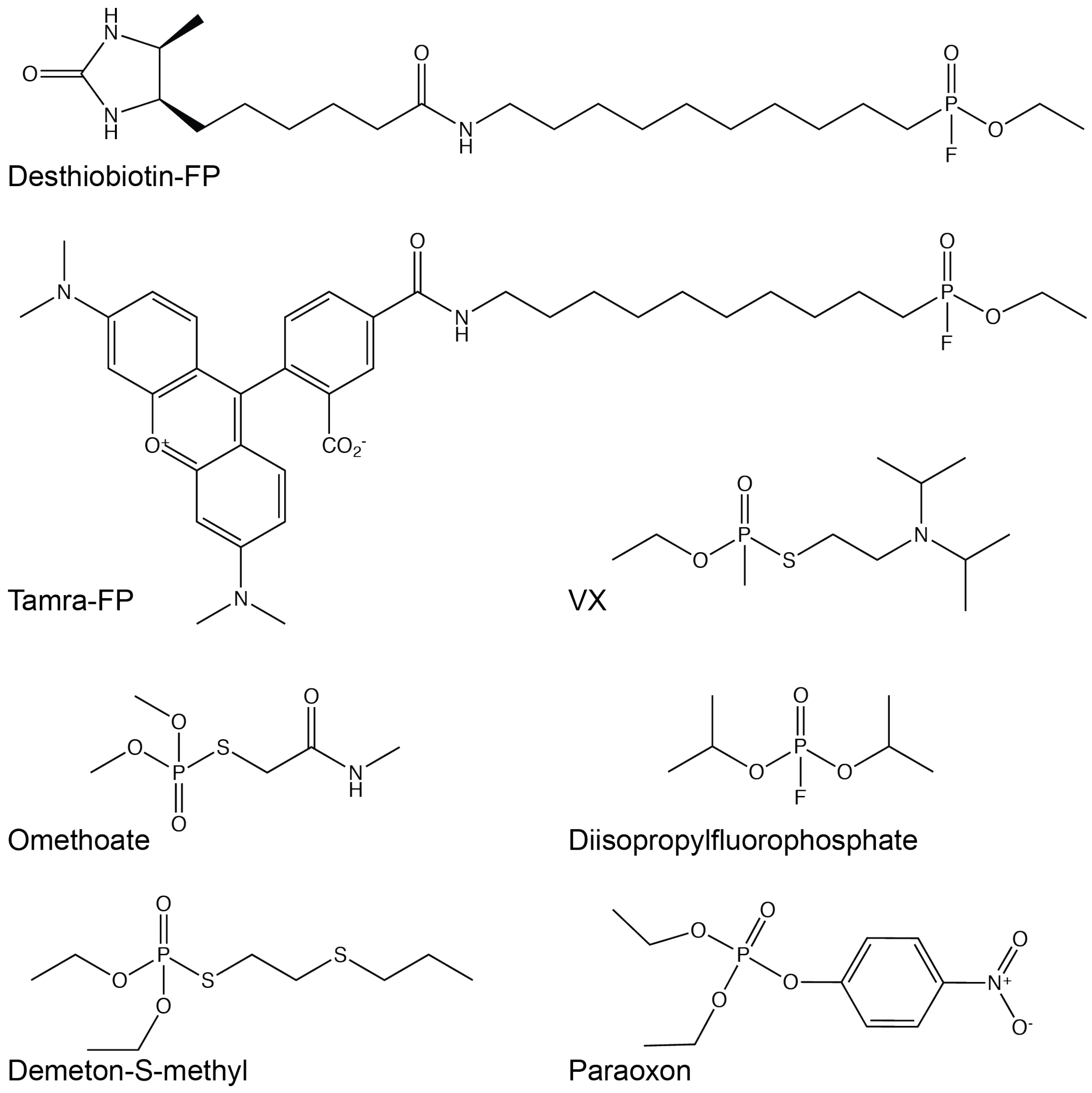

Figure S2. OP structures used in this manuscript. In this figure we show the structure of all of the organophosphates used in this study.

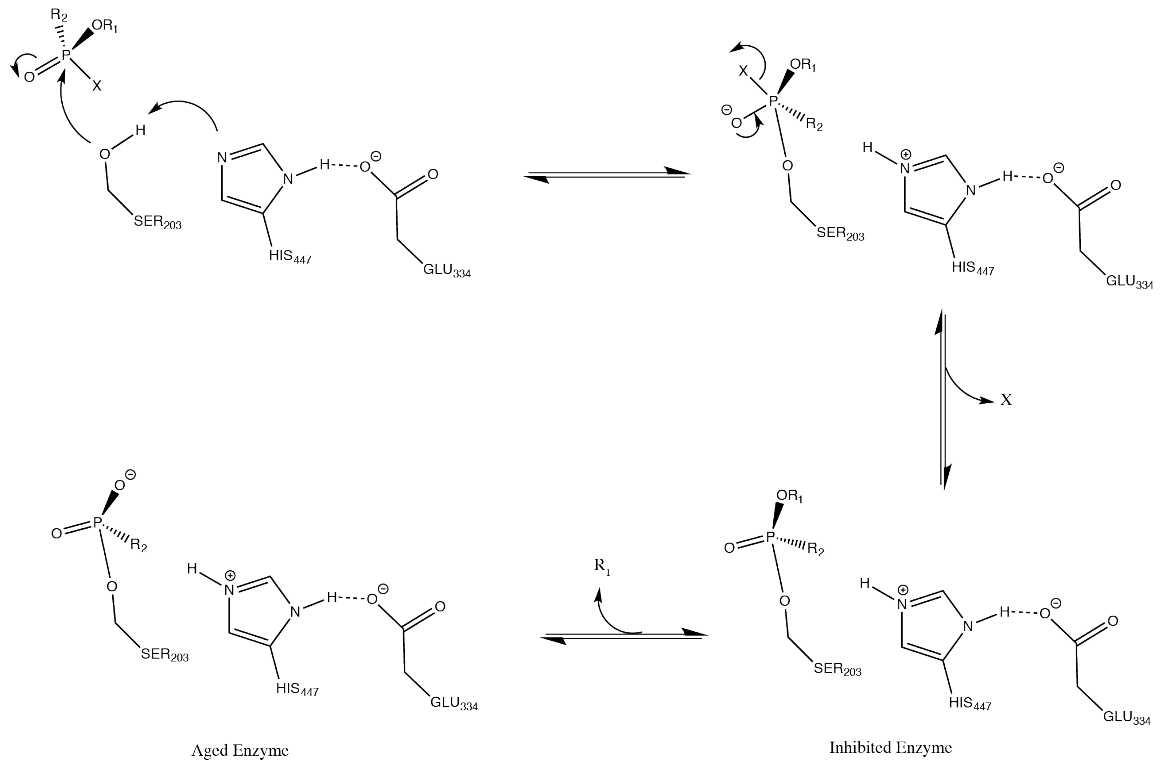

Figure S3. Mechanism of AChE inhibition by organophosphates. In this figure we show the general mechanism of inhibition of AChE by OPs illustrating both the inhibited and aged enzyme construct. The OP leaving group is designated X and is specific to individual OPs.

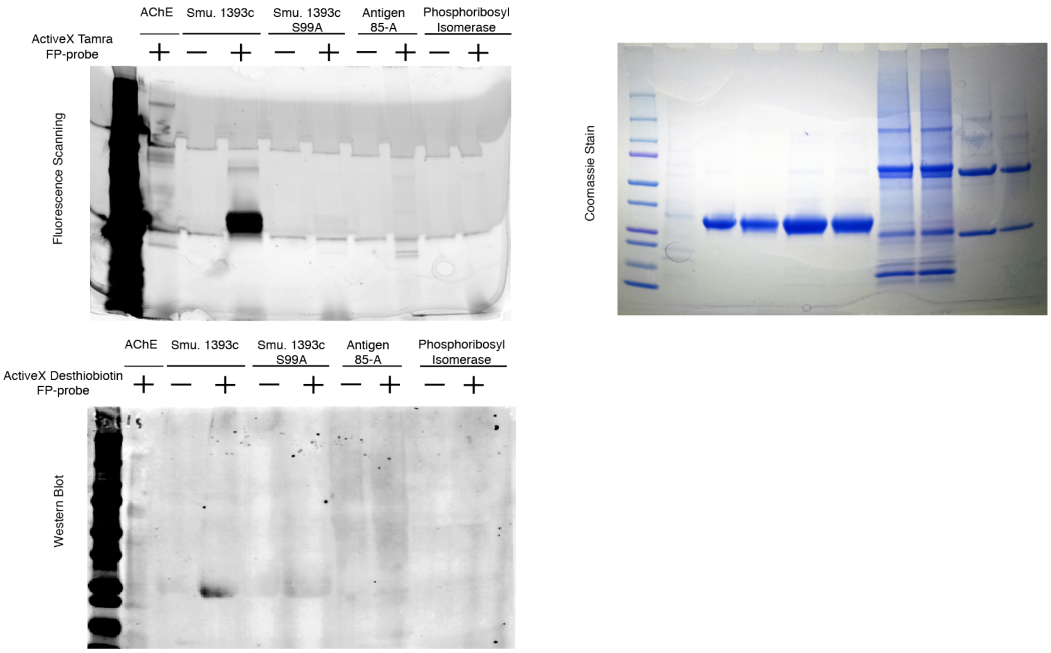

Figure S4. Full fluorescent scanning gel and western blot images. In this figure we show both the fluorescent gel and the western blot in their entirety with accompanying coomassie stained gel.

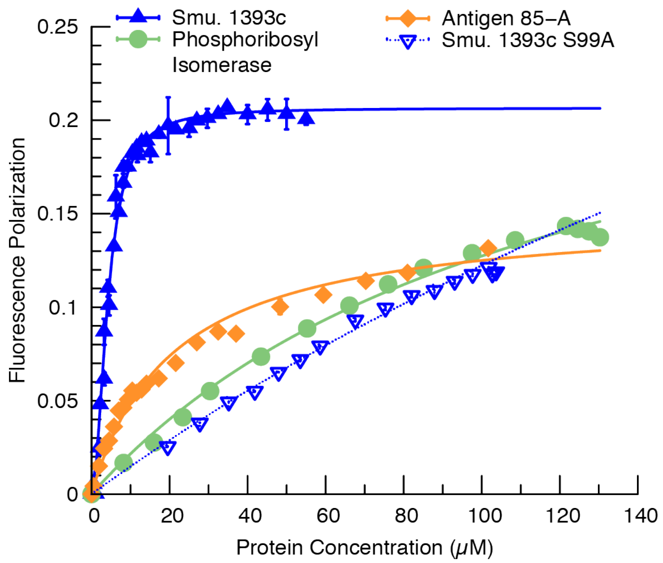

Figure S5. Fluorescence polarization experiment. In this figure we show that candidates Smu. 1393c (blue triangle), antigen 85-A (orange), and phosphoribosyl isomerase (green) interact with the serine hydrolase probe as predicted and confirm the diminished binding of Smu. 1393c S99A (inverted blue triangle).

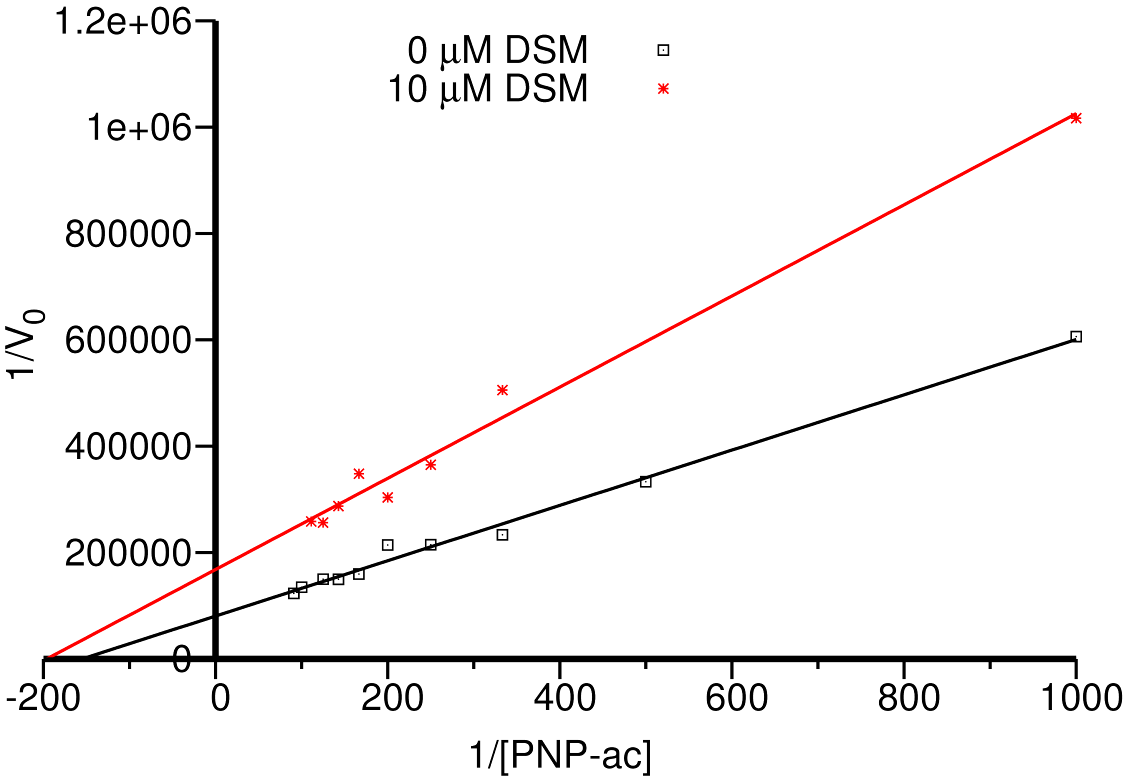

Figure S6. Non-competitive Smu. 1393c inhibition. In this figure we show a Lineweaver-Burk plot of Smu. 1393c activity against PNP-ac with and without inhibitor DSM, illustrating the likelihood of non-competitive inhibition.

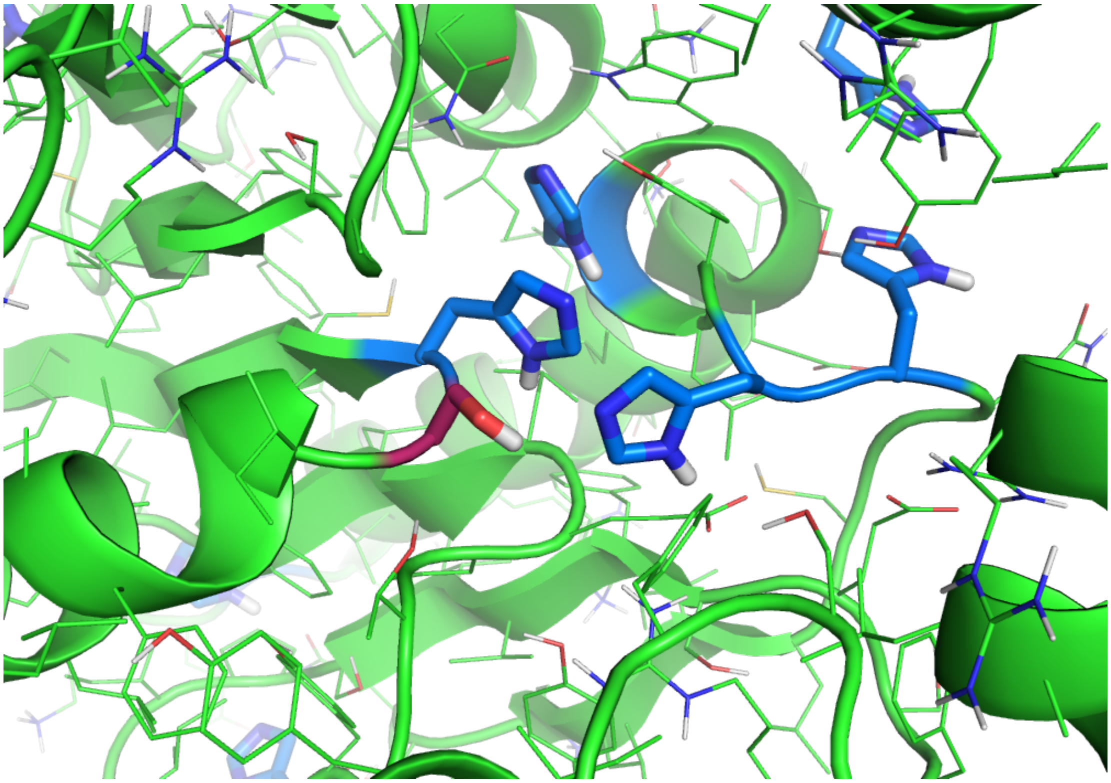

Figure S7. Active site histidine residues of Smu. 1393c. In this figure we show that within the active site cleft of Smu. 1393c there exists a substantial number of histidine residues (blue) near the catalytic serine (S99) (red).

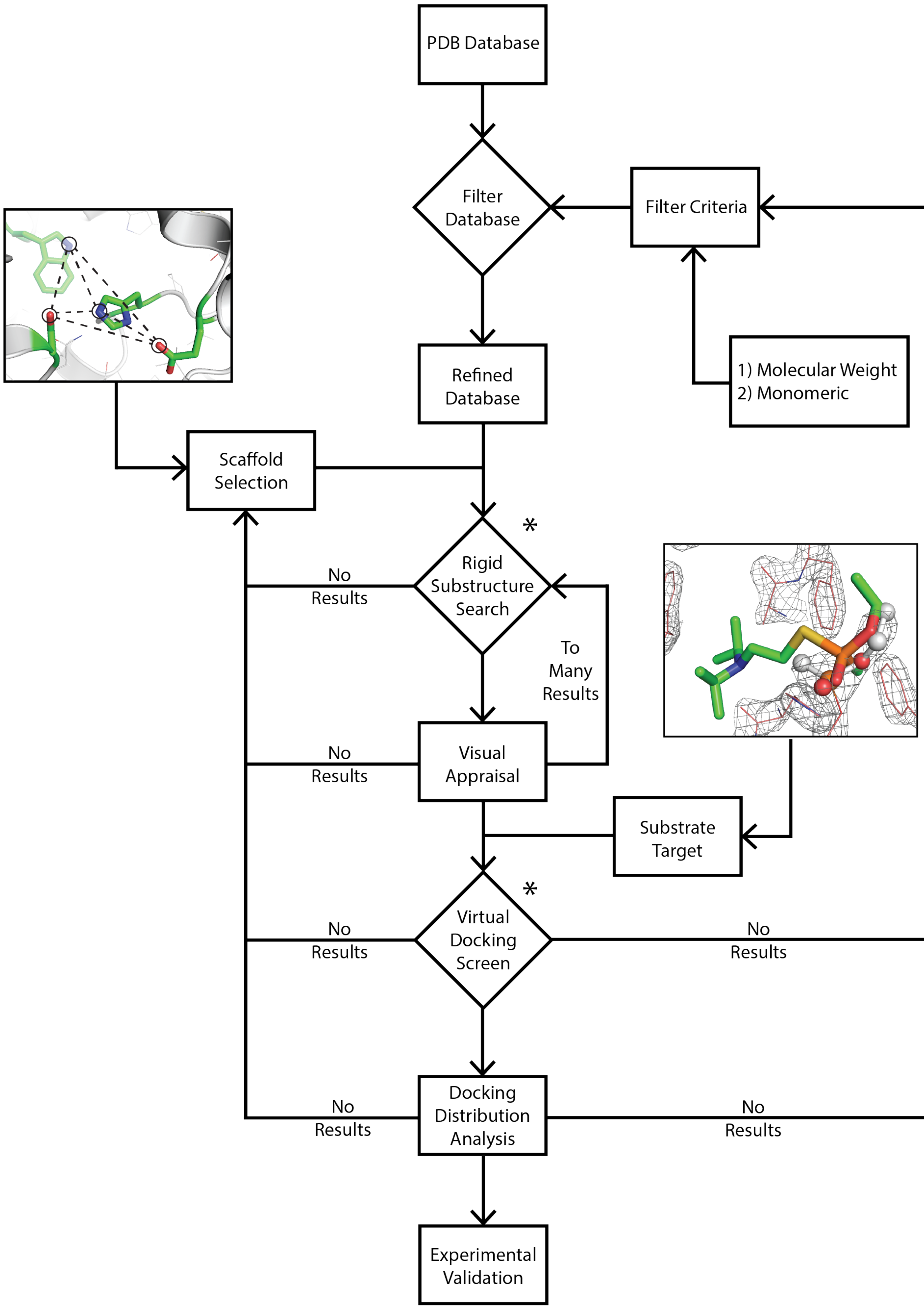

Figure S8. Computational design workflow. In this figure we show the complete computational workflow, including all of the steps and decision points that occur in our computational design. The asterisks indicate where the two algorithms, *Erebus* and *MedusaDock*, are used.

Table S1. Characteristics of potential bioscavenger candidates. For each candidate we list the PDB ID, the scaffold RMSD to the control human BChE, the average ligand RMSD to the control, the top predicted energy returned by *MedusaDock,* the residue length, and the molecular weight.

Candidate	Scaffold RMSD (Å)	Average Ligand RMSD (Å)	Top Docking Energy (kcal/mol)	Residue Length	Molecular Weight (kDa)
1SFR	3.7	6.52	-32.5	304	31.6
2Y85	2.04	5.22	-29.3	244	26.3
4L9A	2.37	5.65	-29.0	292	33.5
3BCN	4.03	8.93	-28.0	209	23.3
2OZ2	4.1	9.67	-27.5	215	23.7
2GHU	4.12	8.11	-26.7	241	27.2
1OCQ	2.03	14.42	-26.6	303	34.6
1H2J	2.77	8.71	-26.0	303	34.8
2V38	2.03	15.62	-25.9	305	34.9
1A3H	2.77	8.91	-25.9	300	33.7
3GI1	4.69	6.61	-25.5	286	32.0
3RJX	2.35	7.29	-25.5	320	37.6
1F29	4.07	9.38	-25.5	215	23.3
1QHZ	4.05	6.37	-25.2	305	34.4
1H5V	2.76	9.4	-24.7	304	35.6
1E5J	2.76	9.06	-24.5	305	35.1
1MEG	6.07	9.97	-23.1	216	23.7
2WHJ	2.04	8.61	-22.9	308	34.7
2OUL	4.15	8.44	-21.6	241	26.9
3BPF	4.03	8.9	-21.2	241	27.6
2BDZ	3.98	9.48	-20.9	214	24.2

Table S2. AChE protection by Smu. 1393c. Kinectic parameters showing positive control (uninhibited AChE) and AChE that is protected by Smu. 1393c from both demeton-S-methyl (DSM) and diisopropylfluorophophate (DFP).

	K_m (mM)	k_{cat} (s^{-1})	k_{cat}/K_m (M^{-1} s^{-1})
AChE	3.05	597.6	2.0×10^5
AChE/Smu. 1393c/DSM	4.43	504	1.1×10^5
AChE/Smu. 1393c/DFP	3.24	456	1.4×10^5

Supplementary Material

Our substructure-matching algorithm *Erebus* requires specific input for reliable results. Our first constructed query is based on the backbone or secondary structure features of AChE with no significant candidates returned. We believe this lack of results is due to the unique topology held by both AChE and BChE, on which key residues are located between two β sheets allowing for higher flexibility, and, thus, multiple substrates. Additionally, it is important to consider any bias introduced based on the query structure, for example, we introduce a bias by choosing residues that typically characterize serine hydrolases' active site (SER203, GLU334, HIS447 and TRP86). As expected from our bias, many of the returned proteins are classified as serine hydrolases, with only a few having unrelated functions, such as phosphoribosyl isomerase.

An additional consideration when using *Erebus* lies in correctly setting the parameters to return sufficient results. In our search, we identify only four of the eight seeded structures (PDB ID's 2X8B, 1P0I, 2XQF, and 2WID). Upon further examination, all of the unreported structures contain an active serine with a covalent modification, which increases the geometric relationships among the key atoms, thus placing the structure outside the *Erebus* preselected RMSD cutoff. A potential solution is a decrease in the sensitivity parameters, allowing a greater variability to be considered a positive match. Unfortunately, this decrease increases the number of returned results from hundreds to thousands. We hold this solution in reserve to be used when there is no identified result from the more stringent search parameters.

MedusaDock is a unique algorithm that considers the flexibilities of both receptor side chains and ligands. The receptor side chains near the binding site can sample all possible rotamers. The docking process consists of two steps, 1) a course docking with rigid-body minimization and 2) a fine-docking step where both side chain and ligand rotamers are considered in the final energy. This method of docking allows *MedusaDock* to successfully predict ~80% of blind pose prediction within 2.5 Å in the CSAR 2011 docking benchmark[1]. When using molecular docking, it is important to determine the number of docking runs necessary to reach proper convergence to the lowest energy pose. This number is system specific and after a convergence study we determine that 100 docking simulations is sufficient for a rapid screen, and 1000 docking simulation to compute the predicted binding energy distributions.

1. Ding, F. & Dokholyan, N. V. Incorporating Backbone Flexibility in MedusaDock Improves Ligand-Binding Pose Prediction in the CSAR2011 Docking Benchmark. *J. Chem. Inf. Model.* **53,** 1871–1879 (2013).
